# Exercise interventions for older people at risk for frailty: A protocol for systematic review and meta-analysis

**DOI:** 10.1097/MD.0000000000025940

**Published:** 2021-05-21

**Authors:** Jianna Zhang, Zhixi Liu, Yi Liu, Lei Ye

**Affiliations:** a West China School of Nursing; b Department of Emergency Medicine, Disaster Medical Center, West China Hospital, Sichuan University, Chengdu, China.

**Keywords:** elderly people, exercise, frailty, meta-analysis, protocol

## Abstract

**Background:**

Frailty is a state of age-related reduced physiological reserve characterized by an increased risk of adverse clinical outcomes. Studies have shown that exercise can improve frailty in older people. However, it remains to be seen which exercises will most improve the fitness of older people with frailty or those at the risk for frailty.

Objective: This protocol aims to determine whether physical exercise can improve frailty in older people, and if which methods are most effective.

**Methods:**

We searched the following databases for relevant articles published between January 1, 2012 and January 1, 2021: PubMed, EMBASE, the Cochrane Library, Wanfang, the China National Knowledge Infrastructure, Clinical Trials Database, and the Science Network. Two independent reviewers will carry out data extraction, discuss and resolve differences, and obtain consensus from the third author. We will select randomized control trials (RCTs) according to the preformulated inclusion criteria. The main outcomes in this study are scores from Fried Frailty Phenotype Criteria; the Frailty Trait Scale–short form; the SHARE Frailty Instrument; the FRAIL scale; the Gérontopôle Frailty Screening Tool; the Clinical Frailty Scale, the Rockwood and Mitnitsky Frailty Index; the Study of Osteoporotic Fractures Index; the Edmonton Frailty Scale; the Fatigue, Resistance, Ambulation, Illness and Loss of Weight Index; the Multidimensional Prognostic Index; the Tilburg Frailty Indicator; PRISMA-7; the Groningen Frailty Indicator; the Sherbrooke Postal Questionnaire; and the Kihon Checklist. Secondary outcomes are muscle strength, gait velocity, stair-climbing power, and level of spontaneous physical activity. If the heterogeneity test shows slight or no statistical heterogeneity, a fixed effects model will be used for data synthesis; otherwise, a random effects model will be used. We will develop a unified data extraction table that includes a number of parameters. The Cochrane Cooperative Bias Risk Tool will be used to evaluate the methodological quality of the selected RCTs. RevMan Manager 5.3 and STATA 14.0 will be used for data analysis if enough RCTs (more than 10) are identified and selected.

**Result:**

The final results will provide information on the effectiveness of intervention programs for frail older adul and further demonstrate which exercise programs are more effective and which methods can significantly improve frailty.

**Conclusion:**

This protocol will contribute to the development of more effective interventions for elderly individuals with frailty.

**Ethics and dissemination:**

This study applies existing literature references; therefore, ethical approval is not required.

**INPLASY registration number:**

INPLASY202130107

## 1 Introduction

The global trend of population aging has become a critical issue for public health, posing enormous challenges to the collective efforts of older adults, health-care professionals, researchers, and policy makers.^[1,2]^ For example, in 2019, the Chinese population alone constituted 18% of the world's population and included some 164.5 million citizens aged 65 years or older and 26 million aged 80 years or older.^[2]^

Frailty is a complex geriatric syndrome that increases vulnerability to stress and frequently results in a decreased physiological reserve in multiple organs, causing limited capacity to maintain homeostasis.^[3]^ At present, there are different degrees of frailty among different elderly populations in various countries around the world.^[4,5]^ Frailty and the risk of frailty are higher in older patients with more health problems, higher body mass indices, and reduced limb and leg strength. Vulnerability is more prevalent in women than in men.^[8]^

Frailness in the elderly is an important factor that can lead to various adverse outcomes, such as waterfall reaction, disability, cognitive decline, hospitalization, rigid thinking, urinary incontinence, prolonged hospitalization, and increased mortality, all of which can seriously affect the body functioning and quality of life of older people. Existing literature has shown that after normal conditions or surgery, older adults with frailty are at higher risk for disability or death than older adults without frailty, and frailty is associated with decreased quality of life in older people.^[6,7,9]^

Infirmity, even physical infirmity, has a large impact on risk for future disability. Certain components of physical frailty, such as slowness, frailness, and weight loss, are strongly associated with disability due to accidents among community-dwelling older adults.^[10]^ Weakness, another aspect of frailty, is related to many factors, including exercise, nutrition, and age. Therefore, exploring factors for preventing and improving frailty in the elderly is of high importance.

Loss of muscle mass may reduce individuals’ independence; when this process becomes chronic, it may lead to frailty. Exercise can increase muscle oxygen content, tolerance, and flexibility; prevent and decrease muscle atrophy; increase muscle mass; enhance muscle strength; improve pace; and improve quality of life and confidence. Results from recent studies indicate positive effects, such as physical functioning, cognition, and psychological well-being, from structured exercise programs during long-term care. At present, therapy for older people with frailty mainly includes endurance training, resistance training, and comprehensive functional training.^[11]^ A combination of strength training and high-intensity interval training improved frailty in 64% of subjects, increased SPPB scores by 3.2 points, and increased muscle strength by 47%.^[13]^ The Lifestyle Interventions and Independence for Elders studies demonstrated that, compared with health education programs, physical activity programs can reduce the risk of major outcomes in a major mobility disorder.^[12]^

Trombetti et al found that a structured, moderate-intensity physical activity program over a 2-year period did not improve frailty in sedentary, community-dwelling older adults.^[17]^ Whether physical exercise can improve frailty in older people is yet to be fully studied. Exercise has been shown to improve frailty in older patients undergoing colorectal surgery,^[18]^ but few targeted studies exist on frailty in sick older adults. Some studies have shown that exercise can improve frailty in older people,^[11,14–16]^ but these do not provide recommendations for the best exercise regimens. The best interventions for addressing frailty among older adults have not yet been fully defined, and the diversity of interventions and outcome measures make the process of determining best practices challenging. Furthermore, additional studies are needed to determine which exercises are best suited, most effective, and safest (in terms of type, setting, duration, frequency, and intensity) for these populations. This project proposes systematic evaluation and meta-analysis to compare exercise modes to determine which are best for improving frailty and reducing risk for frailty in older people.

## 2 Methods and analysis

### 2.1 Eligibility criteria

#### 2.1.1 Types of studies

Randomized controlled trials (RCTs) of exercise interventions for older adults with frailty published in Chinese or English will be included in our review. Studies that are not RCTs but meet the criteria of RCTs are also included.

#### 2.1.2 Types of participants

Studies with participants over 65 years old who could walk without assistance will be included. Studies featuring participants with any of the following will be excluded:

- Visual and hearing impairment interfering with communication or daily activities,
- Cognitive impairment defined as 3-item recall ≤1,
- Functional impairment defined as not able to walk for 5 meter without assistance,
- Suicidal ideation defined as Suicide Subscale of the Mini International Neuropsychiatric Interview ≥6,
- Alcohol abuse disorders active within the year before the study (score ≥2 on the Chinese edition of the Cut down, Annoyed, Guilty, and Eye-opener substance abuse screening tool),
- Organic mental disorders (seizure, brain tumor, brain surgeries), history of schizophrenia, or bipolar diagnosis from a psychiatrist.

#### 2.1.3 Types of interventions

The systematic review and meta-analyses are based on studies in which 1 or more exercise interventions are applied in the experimental group, including single forms of strength exercises.

#### 2.1.4 Primary outcomes

The main outcomes in this study are the scores of the following frailty indices referenced in the literature: Fried Frailty Phenotype Criteria^[19]^; Frailty Trait Scale–short version^[20]^; SHARE Frailty Instrument^[21]^; the FRAIL scale^[22]^; the Gérontopôle Frailty Screening Tool^[31]^; the Clinical Frailty Scale^[25]^; the Rockwood and Mitnitsky Frailty Index^[22]^; the Edmonton Frailty Scale^[23]^; the Fatigue, Resistance, Ambulation, Illness and Loss of weight Index^[24]^; the Multidimensional Prognostic Index^[26]^; the Tilburg Frailty Indicator^[27]^; PRISMA-7^[28]^; the Groningen Frailty Indicator^[29]^; the Sherbrooke Postal Questionnaire^[30]^; and the Kihon Checklist.^[32]^

#### 2.1.5 Secondary outcomes

The secondary outcomes are muscle strength, gait velocity, stair-climbing power, and level of spontaneous physical activity.

#### 2.1.6 Search methods

We will use the following databases: PubMed, Embase, the Cochrane Library, Wanfang, China National Knowledge Infrastructure, Clinical Trials Database, and Science Network as data sources. The following search keywords will be used:

- 1#: “frailty” or “weak”
- 2#: “older people” or “aged” or “elder” or “elderly”
- 3#: “physical exercise” or “exercise” or “training” or “exercise training” or “exercise prescription” or “Tai chi” or “yoga”

These will be combined as “1# and 2# and 3#.”

### 2.2 Data collection and analysis

#### 2.2.1 Selection of studies

**Figure 1:**
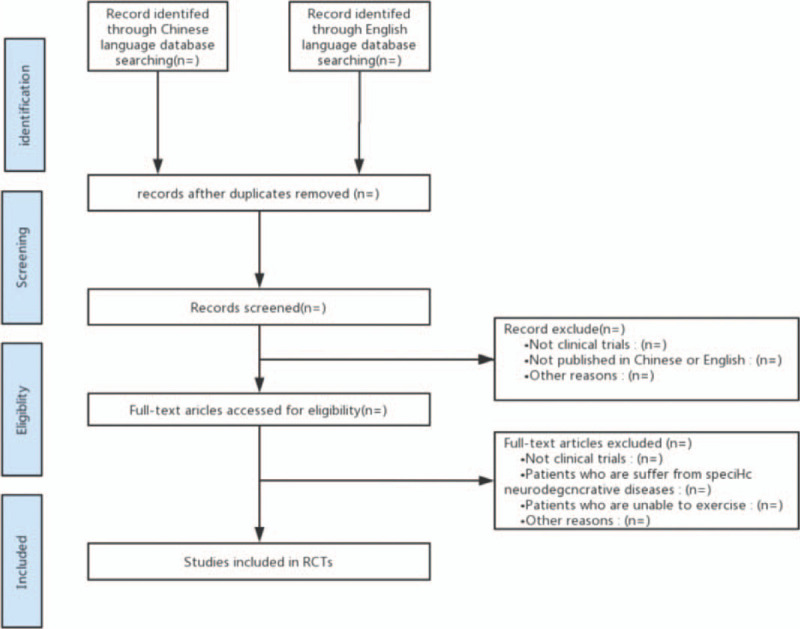
Flow diagram of this study selection.

Two researchers will independently select topics and abstracts to determine the literature that meets the criteria and then read the entire text to further screen the items and record reasons for exclusions. Disagreements will be discussed and decided by consensus, with the intervention of the third researcher, if necessary. Details of study selection and the identification process will be presented in a flow chart (Fig. 1).

#### 2.2.2 Data extraction and management

After study selection, we will conduct a standard data abstraction using a Microsoft Excel 2010 spreadsheet to collect data of interest. One reviewer will extract the following characteristics: first author, year of publication, name of cohort (if any), country in which cohort was located, sample size, mean age, median weight, proportion of female participants, body mass index, race, comorbidities, social function score, smoking status (yes or no), drinking status (yes or no), living status (living alone or living with others), work status (currently working, retired, or never worked), frailty criteria, and the number and percentage of participants according to frailty categories (frailty, prefrailty, and nonfrailty).

#### 2.2.3 Study quality assessment

The Cochrane cooperative risk of bias tool will be used to evaluate the methodological quality of the selected RCTs.

#### 2.2.4 Meta-analyses

RevMan Manager v5.3 (The Cochrane Collaboration, Oxford, UK) and STATA v16.0 (Stata Corporation, College Station, TX) will be used for data analysis.

### 2.3 Sensitivity analysis

If it is possible to conduct a meta-analysis, review management software RevMan 5.3 and STATA 14.0 will be used. Continuous outcomes will be expressed as mean difference with a 95% confidence interval. When the mean difference of the outcomes is large or the unit is different, the standardized mean difference will be used. The heterogeneity test between studies will be assessed using the Q statistic and the *I*^*2*^ statistic. Usually, if *P* > .05, *I*^*2*^ < 50%, heterogeneity is considered low enough to conduct a meta-analysis with a fixed-effect model. If *P* < .05, *I*^*2*^ > 50%, heterogeneity is considered high, and a random effects model will be used. Sensitivity analysis will be used to further reduce heterogeneity by removing studies with high risk of bias or individually omitting each study to explore the sources of heterogeneity. Sources of heterogeneity will be investigated by meta-regression using age, gender, exercise intensity, and duration of exercise as covariates.

### 2.4 Assessment of reporting bias

The *I*^*2*^ statistic and the *χ*^*2*^ test will be used to assess statistical heterogeneity, with *P* < .05 of the *χ*^*2*^ test or *I*^*2*^ > 50% suggesting high statistical heterogeneity among the studies. If the included studies have existing heterogeneity, a random-effects model will be used. Otherwise, we will use a fixed-effects model for calculation.

### 2.5 Subgroup analyses

Subgroup analyses will be performed on physical activity/exercise type (e.g., aerobic exercise, resistance exercise, motor skill training, or mixed training), intensity, frequency, and/or duration, and others. The purpose of subgroup analyses is to determine the best form of physical activity for older people with frailty.

### 2.6 Ethics and dissemination

This project is designed to apply existing literature references; therefore, ethical approval is not required.

## 3 Discussion

Frailty is a geriatric syndrome with a highly complex mechanism.^[33,34]^ It is a complex age-related clinical condition characterized by a decline in physiological capacity across several organ systems, resulting in increased susceptibility to stressors.^[35]^ Engaging in sports and exercise is 1 way to alleviate frailty in older people.^[11]^ Multiple studies have shown that comprehensive training, including resistance exercise and aerobic exercise, can improve frailty in older adults.^[36,37]^ Studies also have shown that a combination of exercises or a single exercise can also improve frailty in these populations.^[38]^

However, no studies have shown which exercise pattern is best for improving frailty in older adults. This project proposes systematic evaluation and meta-analysis to compare existing research to determine the best exercise modes for improving frailty in older people. Lastly, we believe this study may lead to several recommendations for older individuals faced with frailty, such as what exercise methods are best and which exercise intensities are most suitable for older people with frailty. Such recommendations also may be of use to researchers and medical practitioners.

The limitations of the proposed study are intrinsic to this type of analysis, since the credibility of systematic reviews and meta-analyses are strongly affected by the quality of the included studies. Currently, in the literature, scales for evaluating frailty include the Fried phenotype indicators, the Rapid Geriatric Assessment, and the Share-F definition, all of which have different judgments on frailty, thus leading to different research results.^[39]^ Definitive data from large, long-term randomized trials are lacking.

## Author contributions

**Conceptualization:** Jianna Zhang, Zhixi Liu.

**Funding acquisition:** Lei Ye.

**Project administration:** Lei Ye.

**Resources:** Zhixi Liu.

**Writing – original draft:** Jianna Zhang.

**Writing – review & editing:** Jianna Zhang, Yi Liu.
